# Mesenchymal Stem Cells in Premature Ovarian Insufficiency: Mechanisms and Prospects

**DOI:** 10.3389/fcell.2021.718192

**Published:** 2021-08-03

**Authors:** Zhongkang Li, Mingle Zhang, Yanpeng Tian, Qian Li, Xianghua Huang

**Affiliations:** Department of Obstetrics and Gynecology, The Second Hospital of Hebei Medical University, Shijiazhuang, China

**Keywords:** premature ovarian insufficiency, mesenchymal stem cells, therapeutic mechanism, regenerative medicine, ovary

## Abstract

Premature ovarian insufficiency (POI) is a complex endocrine disease that severely affects the physiological and reproductive functions of females. The current conventional clinical treatment methods for POI are characterized by several side effects, and most do not effectively restore the physiological functions of the ovaries. Transplantation of mesenchymal stem cells (MSCs) is a promising regenerative medicine approach, which has received significant attention in the management of POI with high efficacy. Associated pre-clinical and clinical trials are also proceeding orderly. However, the therapeutic mechanisms underlying the MSCs-based treatment are complex and have not been fully elucidated. In brief, proliferation, apoptosis, immunization, autophagy, oxidative stress, and fibrosis of ovarian cells are modulated through paracrine effects after migration of MSCs to the injured ovary. This review summarizes therapeutic mechanisms of MSCs-based treatments in POI and explores their therapeutic potential in clinical practice. Therefore, this review will provide a theoretical basis for further research and clinical application of MSCs in POI.

## Introduction

Premature ovarian insufficiency (POI) is a complex endocrine disease common in women aged below 40 years with global incidence of 1% (Kokcu, 2010; Torrealday et al., 2017; Chon et al., 2021). In most cases, various factors lead to premature exhaustion of primordial follicles pool. This disorder can be caused by autoimmunity, genetic abnormalities, chemotherapy, radiotherapy, or surgery (Kim et al., 2011; Kovanci and Schutt, 2015; Huang et al., 2021). The main symptoms include menstrual disorder, high follicle-stimulating hormone (FSH) and low estrogen levels. Occurrence of POI at a young age leads to harmful effects on reproductive health and may cause sterility. Moreover, estrogen deprivation is associated with sexual dysfunction, low bone density, cardiovascular disease, and severe psychological burden. Although hormone replacement therapy (HRT) is the conventional clinical treatment option for management of POI patients (Sullivan et al., 2016), it has a low efficacy owing to the non-fundamental restoration of ovarian function and severe side effects.

Mesenchymal stem cell (MSC) is a kind of multipotent non-hematopoietic stem cell with low immunogenicity (Pittenger et al., 1999). MSCs have been widely explored as cell-based therapy, and have high potential in managing various diseases by rebuilding homeostasis in inflamed or injured organs and tissues (Levy et al., 2020). MSCs were initially isolated from bone marrow (Caplan, 1991), however, MSCs can also be isolated from multiple tissues, including the umbilical cord, adipose tissue, and placenta (da Silva Meirelles et al., 2006; Hass et al., 2011). Although the initial therapeutic effect was attributed to their multipotency, MSCs also have extensive physiological effects including immunomodulatory activities, preservation of organ homeostasis, and regeneration of injured tissue. Notably, paracrine effectors of their secretome, including cytokines, growth factors, and miRNA, can be transferred to target cells in damaged tissues, resulting in long-term effects (Ranganath et al., 2012; Sajeesh et al., 2020). Several studies have explored efficacy of MSCs-mediated therapy in various diseases owing to their unique characteristics (Martinez-Carrasco et al., 2019; Oliva, 2019; Salado-Manzano et al., 2020). Transplantation of MSCs is a good option for fundamental POI treatment owing to their low immunogenicity, broad sources, and availability. MSCs transplantation is currently widely adopted in studies on POI, which have preliminarily explored the internal mechanisms, including homing (Liu et al., 2014), immunomodulatory, and anti-apoptosis activity (Chen et al., 2018). In brief, after *in vivo* transplantation, MSCs migrate to damaged ovaries where they potentially inhibit release of inflammation-related cytokines, target immune cells to exert immunomodulatory activities, and target damaged tissue cells to regulate proliferation, autophagy, oxidative stress, and fibrosis.

Understanding the molecular and cellular mechanisms underlying MSCs-based treatment of POI is the first step in ensuring safety and efficacy in clinical application of MSCs and for improving quality of MSCs products. Although studies on MSCs-related therapy for POI have explored some internal therapeutic mechanisms, there is no clear correlation between these studies, and the mechanisms presented are disorganized. In addition, a well-conducted systematic and comprehensive summary of these mechanisms is not available. This review will summarize relevant studies on therapeutic mechanism involved in MSCs-mediated treatment of POI, and report limitations in the current studies in this field and provides a basis for future application of MSCs in clinical practice.

## Ovarian Function and Characteristics of POI

The ovary is the most critical female reproductive organ and it performs gametogenic and secretory functions (Brown et al., 2010). A healthy ovary is important for production of sex hormones, which are necessary for the regulation of female growth, menstruation, and modulation of reproductive cycle in the reproductive lifespan (Edson et al., 2009). Physiological or reproductive functions can be impaired in females with dysregulated ovarian function. Granulosa cells (GCs) and oocytes are essential components in the ovarian functioning system, and constitute the main features of follicles. GCs play important roles in maintaining follicular evolution, and secretion of hormones and growth factors that regulate oocytes growth (Maruo et al., 1999). Expressions of hormone receptors in GCs, such as estrogen receptor and follicle-stimulating hormone receptor (FSHR), are important for folliculogenesis and ovulation (Binder et al., 2013). Notably, the number and quality of functional oocytes in the ovary indicate the reproductive potential of females (Practice Committee of the American Society for Reproductive Medicine, 2015). Mature oocytes surrounded by GCs can react to relevant hormones and growth factors (Dewailly et al., 2016), such as FSH and bone morphogenetic proteins during folliculogenesis (Meduri et al., 2002; Dewailly et al., 2016).

Although the cause of POI is unclear in most cases, accelerated apoptosis of GCs and oocytes, blocked follicle maturation, and abnormalities in follicle activation resulting in dysfunction or depletion of ovarian follicles are potential molecular mechanisms of POI (Jin et al., 2012). Therefore, understanding the mechanisms associated with the dysfunction and depletion of the follicular pool can help in development of effective approaches that alleviate ovarian dysfunction. However, the pathophysiology of POI has not been fully elucidated in most cases. A few follicles are activated and develop into mature follicles during menstrual cycle. Moreover, most primordial follicles exist in the dormant state, thus avoiding premature follicular depletion. Pathological state of the ovary, which may be caused by genetic abnormalities, autoimmunity, iatrogenic treatments, and environmental factors, can lead to follicle dysfunction and depletion (De Vos et al., 2010). Approximately 20 ∼ 25% of POI cases are caused by genetic factors, including chromosomal abnormalities and genetic mutations (Jiao et al., 2012). Several related genes have been reported to play a critical role in familial POI, and approximately 14% of cases have a positive POI family history (Bachelot et al., 2009). Further, POI is associated with various autoimmune diseases (Dragojevic-Dikic et al., 2010). Ovarian function damage caused by the autoimmune attack may be the inherent pathogenic factor. However, the exact role of the autoimmune process in ovarian dysfunction should be explored further. Recently, studies report increased cases of iatrogenic POI after treatment of cancer with surgery, radiotherapy, and chemotherapy (De Vos et al., 2010).

In clinical practice, POI diagnosis is accepted in females before 40 years who present with amenorrhea for more than 4 months, with sex hormone deficiencies, and levels of FSH in serum above 40 IU/L (Nelson, 2009). POI can be primary or secondary, as well as ordinary or iatrogenic type (Vujovic et al., 2010). Premature insufficiency of the ovary leads to low levels of estrogen and progesterone. Early ovarian dysfunction increases the danger of osteoporosis, cardiovascular disease, and cognitive decline if it is not treated (Podfigurna-Stopa et al., 2016). Therefore, it is imperative to explore effective therapeutic approaches to manage POI and prevent related complications. European Society for Human Reproduction and Embryology (ESHRE) guidelines state that different causes of POI are suitable for distinguished therapy (Webber et al., 2016). ESHRE recommends use of HRT for treatment of low estrogen symptoms in POI, and HRT can prevent osteoporosis and cardiovascular disease. Although HRT is the most common clinical therapeutic option, it does not effectively restore ovarian function. Other fertility preservation methods and therapeutics, including *in vitro* activation of follicles (Zhai et al., 2016), oocyte cryopreservation (Donnez and Dolmans, 2017), and ovarian tissue transplantation (Mhatre and Mhatre, 2006), have also been explored. However, due to the low efficacy of follicular activation, ethical, technical, and management limitations, these treatment approaches have not been adopted for clinical application. Rapid development of regenerative medicine, such as stem cell transplantation, has high potential in recovery of ovarian function in women with POI. Therefore, scientists in this field should explore the safe and appropriate regenerative medicine-based therapeutic approaches for POI.

## MSCs in Different Diseases

**FIGURE 1:**
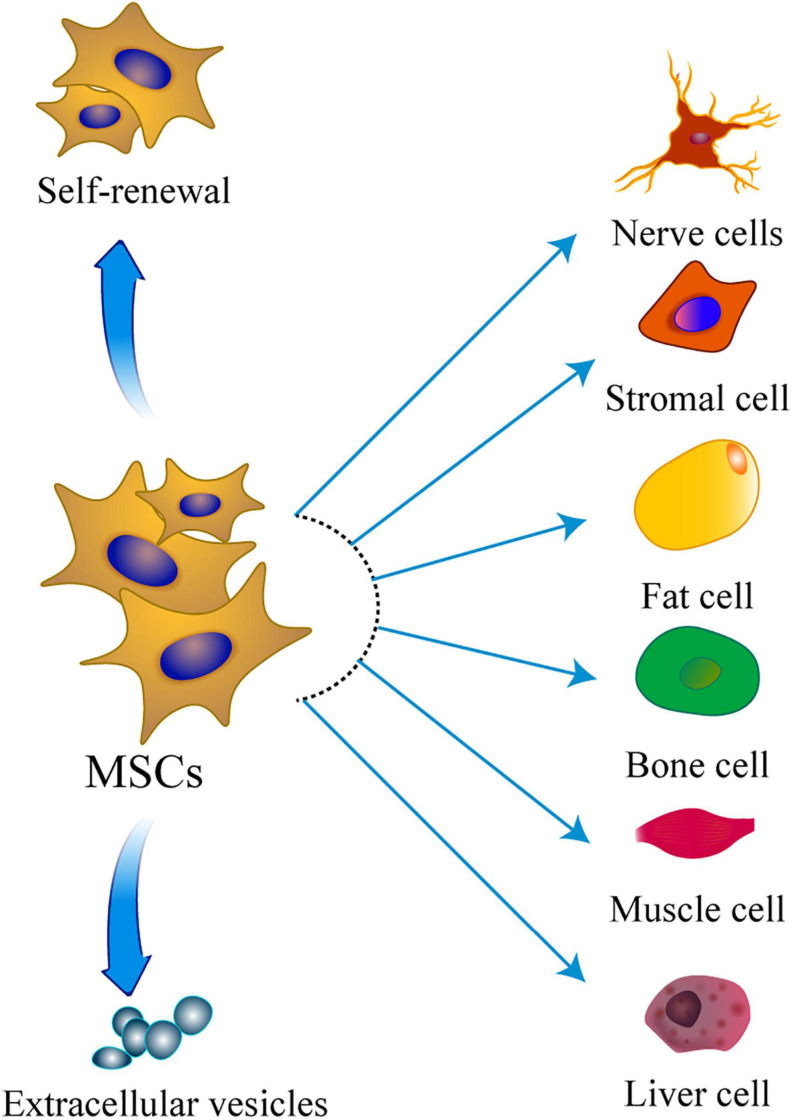
The multipotentiality of MSCs. This figure shows the ability of MSCs to self-renew, secrete extracellular vesicles, and differentiate toward the mesodermal, ectoderm, and endoderm lineage.

Mesenchymal stem cells are a group of different stromal stem cells which can differentiate into various embryonic lineages, mainly the mesodermal lineage (Uccelli et al., 2008; Tang and Lane, 2012; Figure 1). Therefore, MSCs have been widely explored as a promising cell-mediated therapy, with several advantages over other cell origins. MSCs-based therapies have been used to treat a variety of diseases including but not limited to cardiac disease, graft-versus-host disease, and Crohn’s disease. Studies reported critical inherent therapeutic mechanisms by which MSCs demonstrated promising therapeutic benefits in these diseases. Application of MSCs in various diseases is advancing, and shows different progress and different depth. It is important to summarize key findings in application or research processes of MSCs in other diseases which can provide a basis for future treatment of POI.

Reduction of fibrosis, recovery of systolic function, and stimulation of angiogenesis through engraftment and activation of cardiac stem cells, are the main underlying therapeutic mechanisms of treatment of cardiac disease by transplanting MSCs (Karantalis and Hare, 2015). Moreover, reduction of scar tissue and cardioprotection have been reported in clinical and pre-clinical studies. Therefore, to explore potential application of MSCs treatment in POI, studies should evaluate the effects on MSCs on anti-fibrosis, promoting angiogenesis, and activating germline stem cells *in situ*. In graft-versus-host disease, MSCs exert protective effects on the cornea by suppressing T cells (Martinez-Carrasco et al., 2019). Although the specific therapeutic mechanism of MSCs in inflammatory disorders has not been fully elucidated, studies reported that MSCs reduced inflammation owing to their intrinsic immunomodulatory properties (Carvello et al., 2019). MSCs exert immunomodulatory function by homing to target tissues and preventing inflammation of the *in situ* tissue. Therefore, studies should explore immunomodulatory effect of MSCs to understand the mechanism of MSCs-based treatment for immune disease-related POI.

Mesenchymal stem cells exhibit high safety and therapeutic benefits in clinical trials, which has led to approvals of clinical application of MSCs by some institutions. In Korea, an MSC product (Cartistem^®^) was approved for treatment of osteoarthritis, and this novel product is safe and effective (Park et al., 2017). In addition, other various MSC products from different sources have been approved for treatment of various diseases (Zhou et al., 2021).

Mesenchymal stem cells-based treatment of POI shows significant progress as an emerging cell therapy and regenerative medicine technology. Several studies reported that MSCs restore ovarian function; and further studies have explored the underlying therapeutic mechanism. Although MSCs from multiple sources have several advantages and exhibit promising therapeutic effects in management of POI (Esfandyari et al., 2020), the in-depth internal mechanisms should be explored further. Studies of mechanism of MSCs in other diseases can provide a basis for exploring the specific therapeutic mechanisms of MSCs in POI.

## Mechanism of MSCs-Based Treatment for POI

### Migration and Homing of MSCs

The main challenge of MSCs-based therapy is delivery of stem cells to the injury site, a process known as “homing.” Therapeutic efficacy of MSCs is mainly based on their ability to produce paracrine factors to enhance regeneration, therefore, successful delivery to the target damaged organ is essential. Previous studies showed that MSCs can migrate to several organs following intravenous transplantation (Devine et al., 2003). Mechanisms that induce the migration and homing of MSCs are still controversial, however, some hypotheses have been reported. Firstly, unbalanced distribution of MSCs can be attributed to the different degrees of damage in individual organs. Secondly, high number of blood vessels in tissues and organs is correlated with increase in homing of cells. Similar to immune cells, MSCs can exude from blood vessels owing to presence of adhesion molecules on their surface, such as CXCL12 and integrin (Sohni and Verfaillie, 2013). Lastly, MSCs home in response to various chemokines, which potentially attach to their surface receptors (Sordi et al., 2005). Therefore, several factors may regulate homing of MSCs, including tumor necrosis factor α (TNF-α), hepatocyte growth factor (HGF), and fibroblast growth factor (FGF) (Forte et al., 2006).

Studies have explored the homing and migration of MSCs to elucidate the underlying therapeutic mechanism in POI. A previous study administered bone marrow MSCs (BMSCs) through intravenous route and reported that they mainly migrated to the ovarian hilum and medulla, only a few migrated to the cortex, and none migrated to the follicle (Liu et al., 2014). The findings indicated that BMSCs potentially settled in the interstitial of the ovary to restore the function of follicles by their secretion function. Ovarian hilum and medulla are rich in blood vessels, and this may explain the unbalanced distribution of BMSCs in the ovary. In addition, significant distribution of human umbilical cord MSCs (hUCMSCs) in the medulla compared with the cortex has been reported (Jalalie et al., 2019). Moreover, adipose-derived MSCs (ADSCs) were observed engrafted in the thecal layers but not in the follicles (Takehara et al., 2013). It showed that MSCs migrated to the supportive area to promote follicular growth. These findings indicate that the capacity of MSCs to restore damaged ovaries is through a complex process of migration and homing. In another study, Dil-labeled human endometrial stem cells (HuMenSCs) were observed in the GCs’ region of follicles in POI rats (Manshadi et al., 2019). These migrated cells improved folliculogenesis and hormones production in the injured ovary. The findings from this study are not consistent with results from previous studies as they show that MSCs can migrate to follicles. The controversy can be attributed to the diverse characteristics of different cell types, different detection methods and labeling approaches.

Homing and locating in the target organ for a long time are key for efficacy. A previous study reported that hUCMSCs remained alive in the ovary for a long period after transplantation to restore endocrine secretion of POI rat model (Song et al., 2016). Furthermore, GFP-labeled HuMenSCs were detected in the ovarian stroma 2 months after transplantation (Lai et al., 2015). This indicates that after homing to the target organ, part of the MSCs potentially survives longer resulting in long-term therapeutic effect. Notably, homing of MSCs requires mediation of various factors. Gabr et al. (2016) reported that BMSCs were distributed to the ovarian stroma after transplantation, and the ovarian TNF-α and insulin growth factor 1 (IGF-1) levels were increased. The two cytokines can attract MSCs to the injured ovary *in vivo*. MSCs convey several cell adhesion molecules and receptors including CXCL12 and integrin family, and they can induce migration of MSCs to target tissues (Sohni and Verfaillie, 2013).

In summary, after transplantation, MSCs can migrate to injured ovaries to restore ovarian secretory function, follicle formation, and construction in mammalian POI models. Similar to leucocytes, MSCs express various receptors and cell adhesion molecules to improve their migration to target organs, such as injured ovaries. Notably, related chemokines bind to receptors of MSCs to guide their transfer to target tissues. Characteristics of homing of MSCs-related therapy make it an excellent regenerative-based therapy for POI. Following the migration of MSCs to the injured ovary, the proliferation, apoptosis, immunization, and oxidative stress of ovarian cells are regulated by the paracrine effects of MSCs. Therefore, migration is a vital feature for MSCs, and it is an important mechanism for therapeutic efficiency. However, the underlying precise homing mechanisms of MSCs in POI treatment should be explored furthers. These studies of therapeutic mechanisms will provide a basis for further research to improve the treatment efficiency of MSCs in patients with POI.

### Paracrine Effect of MSCs

Previous studies have shown that effective treatment of MSCs is associated with formation of a secretome (defined as the collection of MSCs-derived bioactive factors), which plays a role in restoring ovarian function (Harrell et al., 2019). These bioactive molecules comprise proteins, including IGF, and vascular endothelial growth factor (VEGF); nucleic acids, including micro-RNAs and long non-coding RNAs; extracellular vehicles (EVs), including exosomes, microvesicles, and microparticles (Lotvall et al., 2014).

A previous study reported that MSCs improved ovarian function and structure of ovarian damaged rabbits by secretion of VEGF, which promoted the proliferation of oocytes and GCs (Abd-Allah et al., 2013). In addition, Li et al. (2017) reported that ovarian function was restored following transplantation of hUCMSCs to rats; attributed to induction of secretion of IGF-1, VEGF, and HGF cytokines by hUCMSCs. FGF2 secreted by menstrual-derived stem cells (MenSCs) conferred protective effects on damaged ovaries (Wang et al., 2017). A study on effect of MSCs in natural ovarian aging (NOA) mouse model showed that MSCs-secreted epidermal growth factor (EGF) and HGF were critical factors in restoration of ovarian function by promoting cell proliferation and delaying aging of oocytes (Ding C. et al., 2018). Moreover, most ovarian functions were restored after administration of the combined factors to the NOA mouse model. These findings indicate that independent use of growth factors is a potential therapeutic option for POI management to improve reproductive health. Similarly, hAMSCs pretreated through low-intensity pulsed ultrasound were more effective in reduction of apoptosis in GCs, as this pretreatment increases the function of MSCs to promote secretion of more growth factors (Ling et al., 2017). Growth factors including FGF, IGF-1, HGF, and VEGF have been reported in hAMSCs conditioned media (Ling et al., 2019). Moreover, modification of the ovarian micro-circumstances by paracrine effects is a potential mechanism of transplantation of MSCs. High number of mature oocytes surrounded by GCs have been observed after MSCs transplantation in the micro-circumstances of follicles (Liu et al., 2019).

These findings indicate that paracrine effects of MSCs are beneficial in restoration of ovarian function in POI. In addition, independent use of growth factors is a potential new therapeutic method for POI patients. Research has also revealed that secretions of MSCs potentially modify the ovarian microenvironment. Although migration and secretion characteristics of MSCs promote their curative actions, further studies should be conducted to explore more precise mechanisms. Specifically, the regulation of proliferation, apoptosis, immunization, and oxidative stress of ovarian cells by the paracrine effects of MSCs needs further exploration.

### Proliferation and Anti-apoptotic Effects of MSCs

As mentioned above, high apoptosis rates of GCs and oocytes are correlated with ovarian dysfunction and follicle reduction, which are the critical pathogenesis mechanisms of POI (Santoro, 2003). Therefore, several studies have explored if MSCs-based treatment reduces apoptosis and promotes proliferation of these follicular cells, as well as the therapeutic mechanism.

A previous study reported that UCMSCs restored ovarian functionality by reducing apoptosis of GCs and increasing more oocyte-containing follicles (Wang et al., 2013). A recent study reported that apoptotic effects in ovarian follicles were significantly reduced, numbers of follicles were notably improved, and ovulation recovered remarkably after injection of MSCs into POI mice model (Yoon et al., 2020). Furthermore, MSCs transplantation significantly restored ovarian function by promoting development of follicles and oocytes in cyclophosphamide-damaged ovary (Li et al., 2018). Yang et al. (2019) reported that transplanting hUCMSCs on a collagen scaffold into the ovaries of POI mice promoted GCs proliferation, follicles development, and ovarian angiogenesis. Moreover, hUCMSCs restored ovarian tissue to a normal state by decreasing level of apoptosis and by improving endocrine function of ovaries in POI mice (Shen et al., 2020). A previous study explored the different therapeutic actions between hAMSCs and human amniotic epithelial cells (hAECs). The findings showed that hAMSCs exhibited significant effects in promoting proliferation ratio of human GCs of POI compared with hAECs (Ding et al., 2017). Moreover, human placenta-derived MSCs (hPMSCs) transfer restored ovarian reserve capacity in POI mice through inhibition of excessive apoptosis of GCs and follicular atresia, and the therapeutic efficiency was attributed to promoting expression of anti-Müllerian hormone (AMH) in ovaries (Zhang et al., 2018).

Regulation of expression of related genes, which modulates apoptosis of cells, is an important mechanism for inhibition of apoptosis of GCs and oocytes. Human MSCs increase GCs proliferation, and inhibit their apoptosis by inhibiting expression of Gadd45b protein, which is involved in cell apoptosis (Yan et al., 2019). Similarly, Guo et al. (2019) showed that HuMenSCs combined with the Bushen Tiaochong recipe can rescue ovarian function by decreasing apoptosis of ovarian cells through modulating expression of Gadd45b, pCDC2, and CyclinB1. Moreover, Sun et al. (2013) reported that ADSCs could increase oocyte and follicle number through gene-expression changes and through their paracrine effects. Studies using mice model reported that BMSCs alleviated POI by regulation of genes implicated in apoptosis and proliferation, including p53, Bax, Cyclin D2, and p21 (Bao et al., 2018). Elfayomy et al. (2016) reported that MSCs retrieved existing oocytes by rebuilding normal arrangement of epithelium and improving ovarian niche through modulating expressions of cytokeratin 8/18, transforming growth factor β (TGF-β), and proliferating cell nuclear antigen (PCNA) in the ovary. These genes are critical in management of folliculogenesis and cell proliferation. Furthermore, hUCMSC transplantation regulated the endocrine function of POI rats and decreased expression of apoptosis genes, including caspase-3 (Wang et al., 2020).

Moreover, MSCs can regulate the vitality of oocytes and GCs through various proliferation-related signaling pathways. For instance, hUCMSCs reduced the apoptosis of ovarian cells through regulation of NGF/TrkA signaling pathway (Zheng et al., 2019). Similarly, transplantation of MSCs promoted recovery of ovarian function of autoimmune POI mice by inhibiting the endoplasmic reticulum stress-related IRE1α signaling pathway to reduce apoptosis of GCs (Li et al., 2019). Furthermore, MSCs regulated apoptosis or proliferation of germ stem cells. A previous study reported that BMSCs therapy inhibited germ cell apoptosis, decreased programmed cell death, and restrained DNA damage, which subsequently restore functions of ovarian tissue (Kilic et al., 2014).

In addition to protection of oocytes and GCs multiplication, proliferation of MSCs is important in POI treatment. A previous study detected MSCs after 3 weeks following migration of CD44^+^/CD105^+^ human amniotic fluid MSCs (hAFCs) to the ovaries of POI mice (Liu et al., 2012). Moreover, the hAFCs proliferated and were renewed in the ovary for a long time. Although advances in MSCs transplantation show a prospect of POI treatment, efficiency always is limited, presumably because of the significant apoptosis of transplanted MSCs. Therefore, efficacy can be improved by increasing survival of MSCs. Heat shock (HS) pretreatment is a useful approach for enhancing proliferation and reducing apoptosis of cells. A previous study reported that HS pretreatment promoted proliferation of MSCs (Chen et al., 2018). MSCs exhibited higher adaptation to the microenvironment of POI ovarian tissue and their repair effect was increased following HS pretreatment. Moreover, researchers established the miR-21 lentiviral vector-transfected MSCs, called miR-21-MSCs, which was related to a decreased apoptosis of MSCs (Fu et al., 2017). The miR-21 overexpression in MSCs promoted therapeutic efficacy against POI through downregulation of apoptosis of ovarian cells by targeting phosphatase and tensin homolog (PTEN) and programmed cell death 4 (PDCD4). *In vivo* studies showed that transplantation of miR-21-MSCs was more effective in inhibition of cell apoptosis compared with transplantation of MSCs or miR-21 only.

Rates of proliferation and apoptosis of ovarian cells are related to ovarian function, thus their regulation improves efficacy of MSCs in POI treatment. Therefore, there is a need to explore more detailed mechanisms to improve the therapeutic effect of MSCs and provide a basis for their clinical application.

### Immunomodulatory Effects of MSCs

In MSCs-related therapy, MSCs exert immunomodulatory activities by migrating to affected tissues to inhibit local inflammation. Expressions of various chemokine and cytokine receptors of MSCs promote migration toward inflammatory chemokines and cytokines. Several studies are currently exploring immunomodulatory effects of MSCs (Ben-Ami et al., 2011). MSCs have significant potential in the management of immune-mediated disorders *in vitro* and *in vivo* (Shi et al., 2010, 2018). MSCs confer immunomodulatory actions through several mechanisms. Firstly, MSCs exhibit immunoregulatory characteristics through interaction with immune cells of the innate or adaptive immune systems (Wang et al., 2018). In brief, MSCs regulate the proliferation and function of T-cells (Di Nicola et al., 2002). Then, contact-dependent mode and soluble factors coordinate MSCs-mediated immune regulation (Uccelli et al., 2008). MSCs perform immunoregulatory activities only in the localized target tissue and do not show systemic effects (Devine et al., 2003), thus they do not cause systemic suppression of the immune system.

Studies reported that hUCMSCs can restore the ovarian function of POI mice through regulation of T-helper 1/T-helper 2 (Th1/Th2) cytokines ratio and modulation of number of natural killer cells (Lu et al., 2019). In addition, regulatory T (Treg) cells are important in immune regulation and Th1/Th2 cytokine balance is dysregulated in POI patients. A recent study reported that transplantation of hPMSCs restored ovarian function by regulating levels of Treg cells, TGF-β, and interferon g (IFN-g) cytokines (Yin et al., 2018b). Moreover, the PI3K/Akt signaling pathway mediated the restoration of ovarian function by modulating the balance between Th17/Tc17 and Th17/Treg cells in POI mice model following hPMSCs transplantation (Yin et al., 2018a). Furthermore, a combination of ADSCs with estrogens exerted immune regulation effects by promoting Treg cell proliferation, which ultimately improved the function of injured ovaries (Song et al., 2018).

The autoimmune factor is a common etiology of POI, and the immune-regulating effect of MSCs makes them an effective and promising treatment option for immune-mediated POI. Therefore, future studies should explore the specific mechanisms of immunomodulatory activities of MSCs in POI.

### Autophagy and Oxidative Stress Regulation by MSCs

Autophagy is a cellular degradation process, implicated in elimination of damaged organelles to preserve the normal function of cells (Codogno and Meijer, 2005). It is active in the physiological state or can be activated by cellular stresses, such as oxidative stress (Filomeni et al., 2015). Reactive oxygen species (ROS) is a direct primer that causes oxidative stress, and an early inducer of autophagy (Filomeni et al., 2010). Meanwhile, ROS is a critical inducing factor for ovarian dysfunction (Devine et al., 2012; Liu et al., 2018). Therefore, regulation of autophagy and oxidative stress are essential mechanisms of MSCs-based treatment in POI. Recently, Yin et al. (2020) demonstrated that heme oxygenase-1 (HO-1) gene overexpress in hUCMSCs mediated restoration of the ovarian reserve of POI mice by regulating autophagy through the JNK/Bcl-2 signaling pathway. In addition, hUCMSCs restored ovarian function in POI rats by regulating ROS levels through the autophagy-related AMPK/mTOR pathway (Lu et al., 2020). Huang et al. (2019) showed that fetal liver MSCs exerted antioxidant effects by lowering ROS levels to restore ovary function in POI models. Moreover, transplanted hPMSCs inhibited oxidative stress in injured ovary by modulating the HO-1/HO-2 ratios in ovarian tissues (Seok et al., 2020). Autophagy and oxidative stress are correlated, and the underlying mechanisms of MSCs-based treatment have not been fully explored. Therefore, in-depth studies should be conducted to complete this exploratory work.

### Antifibrotic Effects of MSCs

Fibrosis of the ovary is a major pathological change in POI (Iorio et al., 2014). The TGF-β signal pathway mediated by Smad protein, is essential in physiological process of fibrosis in tissue of multiple organs (Zhang et al., 2017). Currently, researchers intend to explore the antifibrotic effects of MSCs in POI treatment. A previous study showed that MSCs exerted an antifibrotic effect in the injured ovaries of POI rats by regulating the TGF-β1/Smad-3 signaling pathway after transplantation (Cui et al., 2020). Although only a few studies have explored anti-fibrosis effects of MSCs, inhibition and reduction of fibrosis in tissues and organs by MSCs are important mechanisms for the therapeutic effect. Therefore, more studies should explore details on antifibrotic effects of MSCs in reducing ovarian fibrosis.

### Differentiation of MSCs

Mesenchymal stem cells can differentiate into multiple tissue-specific cell types including osteoblasts, adipocytes, cardiomyocytes, and neural cells *in vitro* and *in vivo* (Dawn and Bolli, 2005). Studies reported that differentiation of MSCs may be the underlying mechanism behind tissue restoration (Quarto et al., 2001), however, the specific contribution of MSCs to tissue restoration through differentiation remains elusive (Phinney and Prockop, 2007). A previous study reported that CM-Dil-labeled HuMenSCs were delivered to the ovary and differentiated into GCs within 2 months after transplantation (Noory et al., 2019). Similarly, DiI-labeled HuMenSCs were detected in follicles and analysis showed that they differentiated into GCs (Manshadi et al., 2019). After transplantation into POI models, skin-derived MSCs might differentiate into ovarian stroma cells and activate the germ cell niche (Lai et al., 2014). Abd-Allah et al. (2013) reported that MSCs potentially improved ovarian function of POI rabbits through immediate differentiation into specific ovarian cells.

Although these studies explored the role and mechanism of differentiation of MSCs when they home to the ovary, its significance in restoration and reconstruction of the ovarian function should be explored further. Some studies have shown the possible effects of differentiation; however, more researchers have put forward the opposite opinion. Liu et al. (2014) demonstrated that MSCs do not differentiate into oocytes and GCs. Meanwhile, transplanted MenSCs and ADSCs restored injured ovarian by paracrine effect rather than differentiation into oocytes and GCs (Ling et al., 2017; Wang et al., 2017). Similarly, Ling et al. (2019) demonstrated that the transplanted MSCs did not express the typical markers of oocytes and GCs. Analysis using cell-tracking technique showed that the MSCs did not develop into oocytes or GCs (Wang et al., 2013; Xiao et al., 2014; Zheng et al., 2019).

Direct differentiation may not be a mechanism of MSCs-based therapy and may attract safety concerns during clinical use. In-depth research should be conducted to elucidate the role of differentiation in treatment of POI. Therefore, exploration of the real mechanism of therapeutic effects can light up the way forward in this field.

**FIGURE 2:**
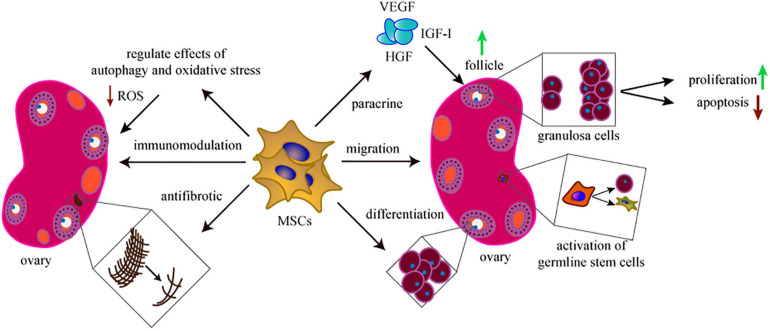
The mechanisms of MSCs in treating POI. The therapeutic mechanisms behind the MSCs-based treatment include migration, proliferation, anti-apoptosis, paracrine, antifibrosis, immunomodulation, antifibrotic, and regulate the effects of autophagy and oxidative stress. Activation of germline stem cells is also involved in this field.

In summary, multiple mechanisms are involved in MSCs-driven treatment of POI. Following the migration of MSCs to the injured ovary, the proliferation, apoptosis, immunization, autophagy, oxidative stress, and fibrosis of ovarian cells are modulated by the paracrine effects of MSCs (Figure 2). The basic mechanisms may be more significant and complex. Therefore, research resources should be used effectively to support the studies of mechanisms for MSCs in POI, to ensure safe and effective application, and transplantation of MSCs.

## New Approaches Based on MSCs (Figure 3)

### Extracellular Vesicles of MSCs

Extracellular vesicles (EVs) are major paracrine effectors derived from MSCs, and have been widely explored in the field of biology and medicine over the past decade (Lotvall et al., 2014). EVs play key roles in cell communication through delivery of numerous proteins and non-coding RNAs (Valadi et al., 2007). Extracellular-RNA (exRNA), such as mRNA and microRNA, carried by EVs, has fundamentally subverted the perception of gene-regulation by potentially regulates the target cells post transcription (Mittelbrunn and Sanchez-Madrid, 2012; Boon and Vickers, 2013; van der Grein and Nolte-‘t Hoen, 2014). Therefore, EVs can signal tissue responses to damage and disease (Abreu et al., 2016). EVs are highly stable *in vivo* compared with other types of particles (Pluchino and Smith, 2019). These characteristics make them advantageous in clinical application of POI. Therefore, cell-free regenerative medicine provides a new low-risk therapy for POI.

**FIGURE 3:**
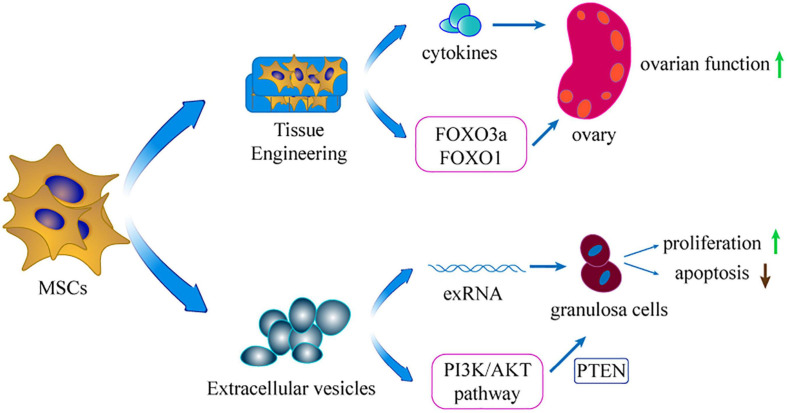
The extension of applicating MSCs. Using extracellular vesicles of MSCs and tissue engineering is the extension application of MSCs in treating POI.

Several studies demonstrated that MSCs-derived EVs comprising biologically active molecules can restore ovarian biological activity and show similar therapeutic effect as transplantation of MSCs. Moreover, the relevant mechanisms of EVs in POI also have been investigated. A recent study demonstrated that EVs could gather around the damaged GCs, and inhibit their apoptosis to restore ovarian secretory function (Zhang et al., 2020). Liu et al. (2020) showed that MSCs-EVs could regulate the PI3K/AKT signaling pathway to enhance ovarian function. Notably, EVs could regulate proliferation-related pathways to modulate the state of ovarian cells, thus restoring ovarian reserve. In addition, exosome miR-144-5p targeted the PTEN (an essential molecule in the PI3K/AKT pathway) to inhibit GCs apoptosis (Yang et al., 2020). Furthermore, overexpression of miR-144-5p in BMSCs administered to POI rats showed higher efficacy compared with using BMSCs alone. Xiao et al. (2016) showed that miR-10a down-regulation in MSCs-derived exosomes repressed the anti-apoptotic effects on damaged GCs *in vitro*. The finding implied that delivering miR-10a is a potential therapy for the preservation of oocytes and GCs in follicles. Therefore, the delivery of exRNA from MSCs-EVs is a potential approach to manage female POI patients.

These findings indicate the potential therapeutic value of MSCs-derived EVs in prompting ovarian function recovery of POI, making the cell-free therapeutic method an advanced approach for clinical application. However, most of the complicated functions and the specific therapeutic mechanism of EVs are unknown. It is imperative to ensure safe usage of this approach in clinical applications to avoid side effects and elucidate the precise mechanisms of ovarian functional recovery.

### Tissue Engineering of MSCs

Tissue engineering is an emerging biotechnology technique that combines cell biology and material science to construct tissues or organs *in vitro* or *in vivo*. Materials such as collagen scaffold can reinforce stem cell attachment, survival, and proliferation. Moreover, MSCs are developed into a 3D-culture model that increases their long-term survival in injured ovaries (Guan et al., 2013). In this model, MSCs can secrete more cytokines compared with use of pure cell culture (Xu et al., 2016). Without tissue engineering, the injected MSCs diffuse rapidly into the neighboring organ and tissue, restricting settlement of external MSCs in the target organ (Suuronen et al., 2006). In addition, inflammation, apoptosis, and ischemia of the transplanted site decrease cell survival in the target organ (Smets et al., 2002). Therefore, application of tissue engineering material for transplantation represents a potential technique to retain the MSCs in the target tissue. Collagen can be acquired from animals and is extensively applied in the tissue engineering field (Ding et al., 2014). A previous study explored the potential ability of collagen scaffolds in protecting transplanted MSCs in the ovary of POI rat model (Su et al., 2016). The findings showed that the collagen scaffold potentially promoted the long-term survival of MSCs in ovaries. Moreover, the transplantation increased the fertility of the POI model. In 2018, the same team used co-culture system integrating ovary-collagen and UCMSCs (collagen/UCMSCs) to explore the therapeutic effects on follicle generation and activation (Ding L. et al., 2018). The findings showed that the collagen/UCMSCs system promoted follicle activation in the ovaries of mice through phosphorylation of FOXO3a and FOXO1. Notably, two POI patients achieved successful clinical pregnancy after transplantation of collagen/UCMSCs or UCMSCs to their failed ovaries. Similarly, another study reported that transplantation of collagen/UCMSCs into the ovaries of mice with POI promoted GCs proliferation and significantly improved ovarian angiogenesis (Yang et al., 2019).

Therefore, combination of tissue engineering material and MSCs for transplantation represents a promising advanced approach for POI treatment with excellent therapeutic potential (Ghahremani-Nasab et al., 2020). However, further studies should explore the inherent mechanism. Additional investigations are needed to settle the clinical application issues, including suitable surgical approaches, appropriate numbers of MSCs, and preparation methods for materials.

## Clinical Applications of MSCs in POI

On the basis of previous investigations and basic mechanistic studies, significant advances in the treatment of ovarian dysfunction can be achieved through clinical trial studies. Application of MSCs in clinical practice was initially used to address osteogenesis imperfecta of children, which showed satisfactory outcomes (Horwitz et al., 1999). Moreover, intravenous infusion of autologous MSCs presented high efficacy through rapid hematopoietic recovery in cancer patients who underwent chemotherapy (Koc et al., 2000). Currently, more than 300 clinical trials on MSCs therapies have been conducted (Levy et al., 2020). Notably, studies report that MSCs exert promising therapeutic effects in some disorders. Pre-clinical and clinical studies are underway on effects of MSCs in POI. The preliminary trial results show that systemic delivery of MSCs in clinical has high therapeutic potential and will bring hope for POI patients. Several clinical trials have been completed and proved effective, therefore, the clinical application of MSCs in POI will be available in the near future. However, uncontrolled use of MSCs may lead to adverse effects (Prockop and Olson, 2007).

Selection of appropriate approaches for application of MSCs in clinical is fundamental for an effective cell-based therapy. Previous studies report that administration of MSCs *in situ* enhances ovarian reserve (Fu et al., 2008; Kilic et al., 2014). Intravenous infusion of MSCs is a straightforward, rapid, and non-aggressive approach. Furthermore, the intravenous approach is beneficial for various injured organs after systemic chemotherapy, therefore, further comparison of the advantages and drawbacks of different treatment methods is should be conducted. Clinical or pre-clinical trials should be prospectively designed to ensure the safety of transplanting MSCs and explore the mechanisms of action and biology of MSCs. Moreover, limited resources should be applied in clinical trials based on the accurately defined mechanisms (Sipp et al., 2018).

## Challenges and Prospects

Although significant advances in MSCs-based treatment for various diseases have been achieved over the past decades, practical application of MSCs still has several challenges. For instance, it is important to verify the safety of transplanting MSCs into ovaries. Fortunately, the safety of administration and application of MSCs gains the highest level of attention. Ensuring safety is a prerequisite for human cell-based therapy. The possible risk of MSCs-based treatment can be attributed to the characteristic of MSCs to suppress immune function and enhance tumor growth (Djouad et al., 2003; Ame-Thomas et al., 2007). A consensus has been reached that there is no threat of malignant transformation when MSCs are cultured *in vitro* (Bernardo et al., 2007). Meanwhile, as mentioned above, MSCs perform immunoregulatory activities only in the target localized tissue and do not cause systemic suppression of the immune function (Devine et al., 2003). Henceforth, ensuring the safety and non-side-effects of MSCs-related treatment is a key focus of basic research and clinical trials in this field. The rich knowledge of MSCs biology can ensure effective application in scientific research and treatment of various diseases, including POI.

Different studies propose distinct methods of isolation, culture, and identification of MSCs for treatment (Kolios and Moodley, 2013). This leads to lack of standardized procedures for the preparation and application of MSCs, which ultimately leads to distinct experimental variables in different experiments and clinical trials. Therefore, it is imperative to develop a systematic standard for MSCs from culture to application (Martin et al., 2019). To circumvent this problem, the International Society for Cellular Therapy proposed a series of fundamental criteria to define human MSCs for both scientific experiments and pre-clinical trials (Dominici et al., 2006). Although these standards are not fully applied in clinical trials, they lay an important foundation for the formulation of more detailed clinical application standards. Development of standards of preparation for MSCs in clinical practice should coincide with the underlying treatment mechanisms, which are dependent on the source of cell, specific disease, and intended use. Scientists propose effective utilization of resources to support the mechanistic studies on MSCs and guide clinical trials to achieve high efficacy in the future (Sipp et al., 2018).

Furthermore, several successful laboratory-based investigations and positive pre-clinical or clinical findings of MSCs-mediated treatment have been reported. An appropriate example is the good therapeutic outcome for complex perianal fistulas of Crohn’s disease using ADSCs (Panes et al., 2016), which was recommended and approved for market authorization by the European Medicines Agency. The positive results from clinics and laboratories motivate researchers to extensively explore the approaches of MSCs-based therapy. Although the road is tortuous, previous promising findings show that the future is bright. MSCs-derived treatment will gradually be applied to manage POI or more multiple complex clinical diseases after standardization of preparation and ensuring safety of application.

## Conclusion

Mesenchymal stem cells are widely used stem cells and studies report that they have great potential for alleviating POI in laboratory-based investigations and pre-clinical or clinical studies in the last decade. Transplantation of MSCs is a great option for fundamental POI treatment owing to their low immunogenicity, broad sources, and availability. Therapeutic effect of MSCs is not modulated by a single factor but comprises a complex biological regulation. Proliferation, apoptosis, immunization, autophagy, oxidative stress, and fibrosis of ovarian cells are modulated through paracrine effects after migration of MSCs to the injured ovary. Tissue engineering and extracellular vesicles are new techniques from MSCs for improving treatment efficacy in POI patients. Developing systematic standards of MSCs from culture to application can improve the safety of MSCs-based applications and avoid side-effects. In summary, MSCs-mediated therapy has promising potential for fundamental restoration of ovarian function in POI patients.

## Author Contributions

ZL and XH were responsible for the concept of the review. ZL was responsible for writing the first draft of the manuscript. ZL and QL made all the figures in this manuscript. YT revised the manuscript critically. XH and MZ were responsible for the critical review of the manuscript. All authors read and approved the final manuscript.

## Conflict of Interest

The authors declare that the research was conducted in the absence of any commercial or financial relationships that could be construed as a potential conflict of interest.

## Publisher’s Note

All claims expressed in this article are solely those of the authors and do not necessarily represent those of their affiliated organizations, or those of the publisher, the editors and the reviewers. Any product that may be evaluated in this article, or claim that may be made by its manufacturer, is not guaranteed or endorsed by the publisher.
